# Knowledge, experience and perceptions regarding Molar-Incisor Hypomineralisation (MIH) amongst Australian and Chilean public oral health care practitioners

**DOI:** 10.1186/s12903-016-0279-8

**Published:** 2016-08-18

**Authors:** K. Gambetta-Tessini, R. Mariño, A. Ghanim, H. Calache, D. J. Manton

**Affiliations:** 1 Oral Health Cooperative Research Centre, Melbourne Dental School, The University of Melbourne, 720 Swanston St, Parkville, VIC 3010 Australia; 2 Faculty of Health, Centre of Population Health Research, Deakin University, 221 Burwood Hwy, Burwood, Melbourne, VIC 3125 Australia

**Keywords:** Molar Incisor Hypomineralisation, Chile, Australia, General dental practitioners, Oral health therapists

## Abstract

**Background:**

Molar-Incisor Hypomineralisation (MIH) is a prevalent developmental defect of tooth enamel associated with a high burden of disease. The present study aimed to survey Australian and Chilean oral health care practitioners (OHCPs) working in public dental facilities and to compare their knowledge, clinical experience and perceptions about MIH. Findings would give insights about how current knowledge has penetrated into OHCPs working into the public systems.

**Methods:**

A mixed-mode survey regarding MIH was carried out amongst Australian and Chilean OHCPs from the public sector. The survey required responses to questions regarding sociodemographics, clinical experience, perceptions, clinical management and preferences for further training. The level of knowledge regarding MIH was determined by Delphi methods for consensus. Data analysis utilised Chi-square, linear and logistic regression models using SPSS Ver. 22.0.

**Results:**

The majority of respondents had observed MIH in their patients (88.6 %) and the level of knowledge regarding MIH was high in Australian participants (*p* = 0.03). Australian respondents felt more confident when diagnosing (OR 8.80, 95 % CI 2.49–31.16) and treating MIH-affected children (OR 4.56, 95 % CI 2.16–9.76) compared to Chilean respondents. Oral health therapists reported higher levels of confidence than Australian general dental practitioners when providing treatment to children with MIH (OR 7.53; 95 % CI 1.95–29.07).

**Conclusions:**

Continuing to update clinical guidelines may help practitioners increase their understanding when diagnosing and treating MIH-affected children. Dissemination of information and awareness regarding MIH is necessary in public clinics, and in particular Chilean general dental practitioners should be alerted to these factors.

## Background

Molar Incisor Hypomineralisation (MIH) is characterised by demarcated enamel hypomineralisation of systemic origin affecting one to four first permanent molars (FPMs) often associated with affected permanent incisors [1]. Similar hypomineralised lesions have been identified in second primary molars and their presence has been reported as a predictive factor for developing MIH [2, 3]. Previously, diagnosis of MIH-affected teeth was complicated by the presence of carious lesions, whereas currently, MIH has become more evident as caries experience decreases in many populations and clinicians’ awareness increases [4, 5]. Children with severe MIH suffer pain and may need several dental visits and referral for specialist treatment. Given its high prevalence (affecting an average 16 % of children worldwide) and burden of disease, MIH brings heavy financial implications for the families and the State.

In order to determine the recognition of MIH as a prevalent condition and as a significant clinical problem, paediatric dental professionals’ perceptions regarding MIH have been determined in many countries [5–11]. The body of information regarding MIH has been concentrated in the paediatric dentistry context and it is not known whether oral health care practitioners (OHCPs), in particular those professionals who are in primary contact with paediatric patients in school dental clinics or community health centres, are familiar with MIH or if adequate information has been provided to them.

In Australia, general oral health care for children in the public sector is mainly provided by Oral Health Therapists (OHTs) followed by GDPs [12]. The scope of practice of OHTs excludes indirect restorations and extraction of permanent teeth, which may be a necessary treatment for severely affected MIH teeth [13, 14]. However, dental authorities have developed a series of clinical guidelines to provide support to all clinicians in the management of compromised first permanent molars, especially those affected by MIH [15]. In Chile, public primary oral health care for children is provided exclusively by GDPs and the public oral health system prioritises six year-old children for comprehensive dental care [16]. Prevention, risk assessment and treatment are based on the Health Ministry Guidelines, which have been edited and improved approximately every three years. However, enamel hypomineralisation has not been included as an independent entity [17].

In both countries, if conditions requiring more complex treatment are diagnosed, affected children are referred to be treated by a paediatric dental specialist. However, a shortage of dental specialists in the public sector has been reported in both countries, particularly in rural areas, however, anecdotally this may be improving [18, 19].

Chilean and Australian oral health systems are to some extent similar; however, their performance has been very different with regard to MIH management. Australia has increased resources focused on MIH (e.g., development of specific guidelines) whilst Chile has increased their efforts in improving general oral health and efforts regarding MIH have remained practically stagnant. A comparison between a heterogeneous public dental workforce from Australia versus a dentist-only staff group from Chile would be of interest, as different dental professionals’ scope of practice and clinical guidelines might have influenced how these professionals are managing MIH-affected children at primary level.

Consequently, the present study aimed to compare the knowledge, clinical experience and perceptions regarding MIH of Australian and Chilean OHCPs from the public dental sector.

## Methods

After approval from the Human Research Ethics Committee from the University of Melbourne and the University of Talca, public dental facilities that provided oral health care for children in Australia (*n* = 393) and family health centres in Chile were invited to participate in this cross-sectional survey (*n* = 435). Facility contact details were identified from each state/country’s health authority website. Data were collected from March to May 2014 in Australia and from October to December 2014 in Chile.

A letter containing the information package and questionnaires was mailed to the centre manager or senior dentist to obtain consent for participation in a mixed-mode survey, giving two options for responding, by post or on-line. In each facility, only one OHCP, the most likely to treat children, was asked to anonymously participate in the study after the authorisation of the centre manager or senior dentist was received. In case the participants chose to complete the survey on-line, the e-mail address of the student researcher (KG) was provided. Participants contacted the student researcher and obtained the survey link. Immediately after sending the link, the received email addresses were deleted. Reminder calls and letters were sent to all the identified centres two and four weeks after the initial mailing.

### Sample estimation

The minimum sample size required to detect an effect size of 0.4, which is considered as a small/medium effect, when testing a mean difference for knowledge, was 100 participants per group (total = 200), with an estimated error of 5 and 80 % power [20]. Mean values and standard deviations were estimated using data collected in a pilot study. This estimated sample size is also sufficient for computing regression models including up to three independent variables.

### Survey instrument and variables

The questionnaire consisted of five sections. In the first section respondents were asked to report sociodemographic information including year and place of dental qualification: a) Australian/Chilean universities, b) Overseas. Completion of post-graduate degrees and type of qualification were also included. The latter was classified as general dental practitioners (GDPs) and oral health therapists (OHTs), which included both OHTs and dental therapists. The second section included questions regarding perceptions and clinical experience with respect to MIH. The third and fourth sections were in relation to knowledge of possible aetiological factors and period of occurrence. With these two sections the knowledge variable was constructed using Delphi methods for consensus. Three experts were invited by email to anonymously weight and score the answers for ten questions about general knowledge regarding MIH diagnosis, prevalence and aetiology [21]. First round of participation was to allocate an individual score to each answer. From second round onwards, an explanation of the answers was necessary and these were shared anonymously between the experts. A total of four rounds were required to achieve consensus. Each answer scored a total of 9 points (Table 1). Subsequently, by summing the ten answers scores, a single continuous variable (i.e., knowledge) was created. The final score ranged from 20 to 60. Higher scores represented higher knowledge regarding MIH.

**Table 1:** Distribution of scores agreed using Delphi methods for each question regarding MIH knowledge and percentage distribution of the (YES) answers between practitioners

		Delphi scores		Percentage distribution of (YES) answers			
Knowledge questions				Total OHCPs	Australia		Chile
		If answered No	If answered Yes		GDPs	OHTs	GDPs
				N (%)	N (%)	N (%)	N (%)
1) Have you been aware that MIH is a developmental defect that differs from fluorosis and hypoplasia?		0	9	198 (86.5)	40 (87.0)	61 (92.4)	97 (82.9)
2) How prevalent do you think MIH might be in your community? (One option chosen)	<5 %	^	1	64 (27.8)	9 (19.1)*	5 (7.6)*	50 (42.7)*
	5–10 %	^	1	72 (31.3)	16 (34.0)*	20 (30.3)*	36 (30.8)*
	10–20 %	^	6	57 (24.8)	10 (21.3)*	25 (37.9)*	22 (18.8)*
	>20 %	^	1	13 (5.7)	3 (6.4)*	8 (12.1)*	2 (1.7)*
	Not sure	^	0	24 (10.4)	9 (19.1)*	8 (12.1)*	7 (6.0)*
3–8) Do you think they are involved in the aetiology of MIH?	3) Genetic factors	4	5	129 (56.6)	27 (57.4)	31 (47.7)	71 (61.2)
	4) Environmental contaminants	4	5	94 (41.2)	22 (46.8)	24 (36.9)	48 (42.4)
	5) Chronic medical conditions affecting mother or child	3	6	136 (59.6)	30 (63.8)**	46 (70.8)**	60 (51.7)**
	6) Acute medical conditions affecting mother or child	3	6	163 (71.5)	35 (74.5)	49 (75.4)	79 (68.1)
	7) Antibiotics or medications	4	5	117 (51.3)	24 (51.1)	34 (52.3)	59 (50.9)
	8) Fluoride exposure	1	8	22 (9.6)	7 (14.9)	5 (7.7)	10 (8.6)
9) During what time/period do you think this insult occurs? (One option chosen)	During pregnancy	^	1	41 (18.1)	3 (6.5)	5 (7.7)	33 (28.4)
	1st year of life	^	3	18 (7.9)	3 (6.5)	6 (9.2)	9 (7.8)
	3rd year of life	^	0	21 (9.3)	6 (13.0)	9 (13.8)	6 (5.2)
	Pregnancy to 1st year of life	^	3	49 (21.6)	10 (21.7)	11 (16.9)	28 (24.1)
	Pregnancy to 3rd year of life	^	2	54 (23.8)	13 (28.3)	20 (30.8)	21 (18.1)
10) Do you think the pattern of caries related to MIH is different from the classical caries pattern?		1 (no) or 1 (not sure)	7	207 (90.0)	42 (89.4)	58 (87.9)	107 (91.5)
Knowledge mean (s.d.)				48.6 (5.9)	48.2 (6.5)	50.0 (5.3)	47.9 (5.9)
Ranges		Min 20	Max 60	29–59	33–59	37–59	29–59
Total sample n =				230	47	66	117
					113		

Results may not add due to missing values

*MIH* molar incisor hypomineralisation, *OHCPs* oral health care practitioners, *GDPs* general dental practitioners, *OHTs* oral health therapists

*Significant differences by *X* ^2^(8) = 40.9, *p* < 0.001

**Significant differences by *X* ^2^(2) 6.46, *p* = 0.04

^ Answer “No” does not apply, as it was analysed as single-choice question

The last section of the questionnaire included questions about practitioners’ confidence in diagnosing and treating MIH, along with preferences in regards to continuing education and views on the necessity for clinical training regarding MIH. The questionnaire was piloted amongst students undertaking the paediatric dentistry specialty training course at Melbourne Dental School to assess applicability and repeatability [8]. The translated Spanish version was revised amongst Chilean students at The University of Melbourne for accuracy of translation.

### Data analysis

The first part of the data analysis provided a descriptive profile of OHCPs and study variables. For the comparison of results Chi-square tests were used. Linear regression models were used to assess the relationship between independent variables (i.e., country, university and postgraduate training) and the knowledge variable in all OHCPs. Binary logistic regression models were computed to test the same independent variables against the confidence in diagnosing and managing MIH. Independent models for Australian practitioners were computed including initial qualification as an additional variable. Independent variables were selected when they met the threshold of *p* < 0.2 in the bivariate analyses. The results were significant at alpha < 0.05. The data were analysed using SPSS Ver. 22.0 (IBM, NY, USA).

## Results

Thirty surveys were returned to sender and a response rate of 29 % (232/798) was achieved. The majority of respondents were female (66.8 %). All Chilean respondents were GDPs, whereas in Australia over half were OHTs (58.4 %). Australian practitioners were significantly older than Chilean dentists (43.5 (s.d. 12.7) years for Australian GDPs and 41.7 (s.d. 12.9) years for OHTs versus 34.1 (s.d. 7.7) years for Chilean practitioners (*p* < 0.001)). Age, gender and the geographic distribution of both samples were compared with available workforce reports.

The overall knowledge mean value was 48.6 (s.d. 5.9; range 29–59) (Table 1). Many Chilean GDPs (42.7 %) perceived that the prevalence of MIH in their communities is less than 5 % (*p* < 0.001). A vast majority (90 %) recognised a different pattern in the clinical presentation of carious lesions related to MIH which did not conforming to the usual caries pattern.

The vast majority of respondents had observed MIH in their patients (88.6 %), and this varied between countries (*p* < 0.001) (Table 2). The most common defects seen by OHCPs were yellow/brown coloured opacities (64.9 %). The majority of respondents in both countries had encountered hypomineralised lesions in permanent teeth other than FPMs (63.9 %). Interestingly, Chilean respondents had not noticed hypomineralised second permanent molars (*p* < 0.001).

**Table 2:** Perceptions, clinical experience and continuing education aspects in OHCPs

Questions		Total OHCPs	Australia		Chile
			GDPs	OHTs	GDPs
		N (%)	N (%)	N (%)	N (%)
Do you encounter teeth with MIH in your practice? (YES)		203 (88.6)	44 (95.7)**	66 (100)**	93 (79.5)**
In what other permanent teeth have you encountered MIH-like defects?	Premolars	71 (66.4)	12 (63.2)**	16 (47.1)**	43 (79.6)**
	Second permanent molars	16 (15.0)	5 (26.3)**	11 (32.4)**	0**
	Canines	20 (18.6)	2 (10.5)**	7 (20.5)**	11 (20.4)**
Do you notice these defects in the primary dentition? (YES)		200 (90.1)	41 (89.1)*	64 (98.5)*	95 (85.6)*
Do you feel the incidence has increased in the period of your practice? (YES)		95 (68.8)	22 (84.6)*	40 (81.6)*	33 (52.4)*
Would you refer a child who has signs of MIH to a paediatric specialist for treatment?	Yes or when possible	129 (56.6)	37 (78.7)**	45 (69.2)**	47 (40.5)**
	No	99 (43.4)	10 (21.3)**	20 (30.8)**	69 (59.5)**
What type of restorative material do you often use to treat these teeth? (Only those YES answers are shown)	Amalgam and other direct materials	18 (7.9)	3 (6.4)	3 (4.6)	12 (10.3)
	CR exclusively	10 (4.4)	1 (2.1)	0	9 (7.8)
	GIC exclusively	63 (27.6)	10 (21.3)	23 (35.4)	30 (25.9)
	RMGIC exclusively	14 (6.1)	2 (4.3)	1 (1.5)	11 (9.5)
	CR, GIC and RMGIC combined	72 (31.6)	6 (12.8)*	18 (27.7)*	48 (41.4)*
	PC combined with direct materials	43 (18.9)	25 (53.2)**	16 (24.6)**	2 (1.7)**
	Others, e.g., cast rest, extractions and fluoride	8 (3.5)	0	4 (6.2)	4 (3.4)
Are you receiving any information on MIH? (YES)		117 (51.5)	22 (46.8)*	45 (68.2)*	50 (43.9)*
Total		230	47	66	117
			113		

Results may not add due to missing values

*OHCPs* oral health care practitioners, *GDPs* general dental practitioners, *OHTs* oral health therapists, *MIH* molar incisor hypomineralisation, *CR* composite resin, *GIC* glass ionomer cements, *RMGIC* resin modified glass ionomer cements, *PC* preformed crowns

**Significant differences by *X* ^2^ *p* < 0.001, *significant differences by *X* ^2^ *p* < 0.05

Chilean participants observed hypomineralised lesions in the primary dentition at a lower frequency (85.6 %) than Australians (*p* = 0.02). The vast majority of the Australian GDPs (84.6 %) and OHTs (81.6 %) reported that they perceived an increased incidence of MIH during their professional lifetime (Table 2). MIH represented a clinical problem (95.7 %) in both countries and all practitioners reported that it would be worthwhile investigating the MIH prevalence in their communities (93.9 %).

The majority of Chilean GDPs (59.5 %) would not refer patients with signs of MIH to a paediatric dentist, whereas the major proportion of Australian GDPs (78.7 %) and OHTs (69.2 %) would always or when possible do this (*p* < 0.001). Preferred treatment options are presented in Table 2. Preformed metal crowns (PMCs) in combination with other direct materials were used sparingly by Chilean respondents (1.7 %), whereas the majority of Australian GDPs (53.2 %) and 27.7 % of OHTs preferred them as a treatment alternative (*p* = 0.001). A larger number of Chilean practitioners considered insufficient training (38.8 %) and information (56.1 %) about MIH as an important barrier to providing treatment (Table 2).

The linear regression model for knowledge indicated that those who completed a postgraduate course (*p* = 0.01) and Australian OHCPs (*p* = 0.03) reported higher knowledge scores (Table 3).

**Table 3:** Multivariate regression analysis for Knowledge variable in all OHCPs

Independent variables		B	SEB	Beta	P value
Constant		49.16	0.86		<0.001
Postgraduate course	1 = Yes	2.28	0.92	0.17	0.01
	0 = No				
How long practice	In years	−0.12	0.04	−0.02	0.75
Country	1 = Chile	−1.90	0.88	−0.16	0.03
	0 = Australia				

F(3,222) = 3.0; *p* = 0.03; *R* ^2^ = 4 %

The logistic regression model demonstrated that Australian OHCPs (OR 8.80) and graduates from local universities (OR 6.99) were more likely to be confident in diagnosing MIH compared to Chilean participants and graduates from overseas (Table 4).

**Table 4:** Sociodemographic variables and OHCPs’ confidence in diagnosing and treating MIH-affected children

Independent variables		Unconfident when diagnosing	Confident when diagnosing	Adjusted odds ratio	95 % CI	P value	Unconfident when treating	Confident when treating	Adjusted odds ratio	95 % CI	P value
		*n* = 24 (%)	*n* = 182 (%)								
							*n* = 63 (%)	*n* = 165 (%)			
Country	Chile	16 (17.0)	78 (83.0)	^			44 (37.9)	72 (62.1)	^		
	Australia	8 (7.1)	104 (92.9)	8.80	2.49–31.16	0.001	19 (17.0)	93 (83.0)	4.56	2.16–9.76	<0.001
University	Overseas	9 (19.6)	37 (80.4)	^			12 (26.1)	34 (73.9)			
	Australian/Chilean	14 (9.1)	140 (90.9)	6.99	2.04–23.95	0.002	48 (27.1)	129 (72.9)	1.93	0.81–4.60	0.14
Postgraduate training	No	20 (13.0)	134 (87.0)	^			47 (28.0)	121 (72.0)	^		
	Yes	4 (8.0)	46 (92.0)	2.69	0.79–9.09	0.11	15 (25.9)	43 (74.1)	1.72	0.82–3.59	0.15
		*X* ^2^(3) = 18.52; *p* < 0.001, Nagelkerke R square = 17 %, 90.5 % of the cases.					*X* ^2^(3) = 18.20; *p* < 0.001, Nagelkerke R square = 11 %, 73.9 % of the cases.				

The second model (Table 4) conducted to evaluate the correlation of selected variables with the level of certainty when providing treatment to MIH-affected children, indicated that Chilean OHCPs had decreased likelihood of being confident when referenced to Australian respondents (OR 4.56).

For Australian OHCPs exclusively, OHTs reported feeling seven times more confident (OR 7.53) when treating MIH patients compared to Australian GDPs (*p* = 0.02).

## Discussion

In the present study it was more likely that Chilean OHCPs may be less aware about the presence of MIH compared to Australian OHCPs. As a starting point and following the Australian example, the inclusion of up-to-date international evidence in the guidelines, particularly in regards to MIH definition, prevalence, aetiology and clinical management, may support public practitioners in their decisions and increase their awareness and understanding about MIH [15]. In Australia and New Zealand, the available reports are from clinicians with an interest in paediatric dentistry showing high levels of awareness of MIH, with their general knowledge being similar to that of Australian OHCPs from the present study [5].

The majority of participants had encountered MIH in their practices and yellow/brown opacities were the most frequent presentation. This result is consistent with previous reports from Australia, Iraq and Malaysia [5, 6, 9]. However, Chilean respondents reported observing this defect less frequently and perceived lower prevalence (<5 %) than Australian practitioners. This may reflect a low awareness level regarding prevalence of MIH in the Chilean population, in spite of the existing evidence from Chilean studies indicating a 16.8 % prevalence [22]. Surprisingly, an overwhelming majority of practitioners (more than 90 %) from both countries recommended that investigating the prevalence of MIH would be worthwhile. These results support the fact that practitioners in the public sector are still uncertain about the prevalence of MIH in their communities [5, 7].

The difference in prevalence reported between countries could also be associated with a lower caries experience in Australia (DMFT of 1.05 in 12 year-old children) compared to Chile (DMFT of 1.9 in 12 year-old children) [23, 24]. This may mask the presence of MIH [4]. Unfortunately, it is not possible to corroborate this hypothesis, as current epidemiological information of MIH and its association with dental caries is scarce in these communities. Considering that a large number of participants confirmed the atypical presentation of carious lesions related to MIH, the present study supports the association between carious lesions and MIH in terms of lesion severity, with a greater prevalence of dental caries in MIH-affected children [25].

Recently, some similar defects to MIH have been reported in the literature, in particular the presence of enamel hypomineralisation in the primary dentition also associated with an increased risk of developing MIH [2, 3]. The majority of respondents in the present study reported observing MIH-like defects in the primary dentition, with Australian perception higher than Chilean. The different perceptions of the presence of hypomineralisation in primary and permanent dentitions could be associated with the level of knowledge that the dental community possesses in relation to these defects. The assessment of knowledge in the present study demonstrated that Australian OHCPs and, not surprisingly, those who had completed a postgraduate course reported higher MIH knowledge. These results may be explained by the fact that in Australia, there are actively organised initiatives for promoting and disseminating information about the impact of developmental defects and its associated cost for health authorities, societies and families, such as the D3 website (http://www.thed3group.org/) [26]. This information could be translated into other languages to reach different populations.

Statistical analyses demonstrated that OHTs reported feeling more confident when providing care for children with MIH than Australian GDPs. This may be explained due to the scope of practice of dental therapists, which has been historically directed to child oral health [27]. The majority of OHTs also reported receiving more information regarding MIH, which also increases knowledge and awareness. Nonetheless, a large number of OHTs mainly rely on GIC for restorative care exclusively (35.4 %) and similar results were reported previously [5]. These results may be controversial, as the use of GIC for large areas affected by hypomineralisation is considered as an interim treatment by many and advocated to reduce sensitivity and to prevent PEB in an attempt to ‘stabilise’ the tooth so that it can be definitively restored at a later date [14]. Training and competencies of OHTs are not sufficient to provide all restorative care necessary in severely MIH-affected children; however, they feel a high level of confidence when diagnosing and treating affected children and most importantly, they refer them to specialists for further treatment in the early stages of the condition. Early diagnosis and prevention of breakdown are key elements in managing MIH [28].

Medium-term and long-term treatment options for MIH teeth should include direct resin composite restorations, cast restorations, extractions and placement of PMCs [14]. Yet, only 1.7 % of Chilean respondents reported using PMCs compared to the majority of Australian GDPs. These results may reflect the difference in the training received at university. Placement of PMCs is part of the dental course in Australian universities, yet in Chile, it is only part of the curriculum in postgraduate paediatric training courses.

Continuing education programs might disseminate MIH knowledge and clinical management amongst dental professionals in both countries to increase confidence when diagnosing and treating children with MIH, especially when over 90 % of practitioners in both countries desired further training especially on MIH treatment modalities. It should be ensured that these programs are accessible to GDPs working in the public sector. For example, in Victoria (Australia), a continuing professional development program has been designed for OHCPs to enhance their clinical practice in the use of stainless steel crowns, albeit in primary molars principally [29]. This is contrary to Chile, where continuing education programs are not compulsory requirements for professional practice registration.

Insufficient training and lack of information regarding current evidence about MIH may affect the management of children with MIH in the public sector setting. It is expected that dental professionals develop the ability to have lifelong learning to search out answers and solve problems using the evidence. However, a large number of OHCPs in the present study reported not receiving adequate information. This situation appears worse in other countries, such as Malaysia where only 7 % of GDPs reported that they have received information regarding MIH [9]. It is important that health authorities consistently and effectively spread scientific evidence and provide clinical management guidance that OHCPs working for the public dental clinics can access. This may help to decrease the treatment burden associated with MIH in the future [9]. It is essential to expand the availability of MIH information not only to those professionals who are specialists, but also to those practitioners who are in the position to diagnose this condition initially.

The present findings should be considered in the context of the study’s limitations. The linear model explained very small percentage of the variation of knowledge, which indicates that many other variables not included in the present study may affect OHCPs’ knowledge regarding MIH. For example, the critical use of the guidelines was not assessed amongst participants. Therefore, it is unclear if the available guidelines facilitate a more cost-effective care and aid the practitioners in their clinical decisions. However, guidelines will certainly not replace the clinician’s judgement and the patient’s personal characteristics [30]. Some Australian centres could have been excluded from this investigation, as health authority websites were reviewed but not all of them provided the full list of available centres. A relative low response rate of 29 % was obtained and this fact limits the generalisation of the results. In spite of that, according to the sample calculation, the number of participants (*n* = 232) is sufficient to give enough power to provide representative results. The self-reported nature of the questionnaire may also increase the possibility of response bias. Nevertheless, the present study provides baseline data for further investigation on the topic in both countries.

## Conclusions

Molar Incisor Hypomineralisation is a prevalent condition encountered by OHCPs in Australian and Chilean public dental clinics. Variations between countries existed particularly in terms of prevalence, aspects of clinical management and perceptions regarding MIH. Chilean respondents reported lower knowledge scores and confidence than Australian OHCPs. Inclusion of up-to-date information about MIH in the clinical guidelines or implementation of accurate continuing development programs may help practitioners to increase their understanding when diagnosing and treating MIH-affected children. More information regarding this topic seems necessary in both countries, and in particular to be directed to GDPs working at the public sector.

## Abbreviations

GDPs: General dental practitioners
GICs: Glass ionomer cements
MIH: Molar incisor hypomineralisation
OHCPs: Oral health care practitioners
OHTs: Oral health therapists
PMCs: Preformed metal crowns
